# Effectiveness of Coenzyme Q10 Supplementation for Type 2 Diabetes Mellitus: A Systematic Review and Meta-Analysis

**DOI:** 10.1155/2018/6484839

**Published:** 2018-09-16

**Authors:** Shi-ying Zhang, Kai-lin Yang, Liu-ting Zeng, Xiao-he Wu, Hui-yong Huang

**Affiliations:** ^1^Hunan University of Chinese Medicine, Changsha 410208, China; ^2^Jiangxi Provincial People's Hospital, Nanchang 330006, China

## Abstract

**Objective:**

To evaluate the effectiveness and safety of coenzyme Q10 for patients with type 2 diabetes mellitus (T2DM).

**Methods:**

Data from randomized controlled trials were obtained to assess the effects of coenzyme Q10 versus placebo or western medicine on patients with T2DM. The study's registration number is CRD42018088474. The primary outcomes included glycosylated hemoglobin, fasting blood glucose, and fasting insulin.

**Result:**

Thirteen trials involving 765 patients were included. Compared with the control group, coenzyme Q10 may decrease the HbA1c (WMD −0.29; 95% CI −0.54, −0.03; *P* = 0.03) and the fasting blood glucose (WMD −11.21; 95% CI −18.99, −3.43; *P* = 0.005). For fasting insulin, there is also not strong evidence that confirms which one is better because there was no statistical difference (WMD −0.48; 95% CI −2.54, 1.57; *P* = 0.65).

**Conclusion:**

Based on current evidence, coenzyme Q10 may assist glycemic control, decrease TG, and improve HDL-C in patients with T2DM.

## 1. Introduction

Diabetes is one of the major global health issues. According to the World Health Organization, in 2014, about 422 million adults were suffering from diabetes. In the US, more than 9% of the US population were affected by diabetes [1–3]. In the global population of diabetes, 90%–95% are with type 2 diabetes mellitus (T2DM). Since 1980, the global prevalence of diabetes in adults has nearly doubled. Diabetes caused 1.5 million deaths in 2012 as it relates to increased risk of cardiovascular and other diseases [4, 5]. And according to estimates of the Diabetes Federation Diabetes Atlas, the number of patients with diabetes in 2035 will reach 592 million [6]. Patients with T2DM are at high risk of developing hyperglycemia-related cardiovascular complications such as heart disease, hypertension, stroke, retinopathy, and nephropathy. In the US, disability, loss of work, and premature death caused by diabetes have resulted in a huge direct and indirect medical costs [2]. Without effective prevention and management programs, a further significant increase in diabetes will have grave consequences on the health and lifespan of the world population [6]. At present, the treatment of diabetes is still lacking etiological treatment; the treatment of diabetes is mainly achieved through diabetes management including healthy eating, weight control, appropriate physical activity, antiglycemic medications, and multifactorial risk reduction [7].

T2DM occurs when the organism fails to respond to the increased blood glucose caused by the impaired *β* cell secretion and/or insulin resistance [1, 8]. Therefore, T2DM is a complex chronic disease. Oxidative stress and insulin resistance are recognized pathogenic mechanisms in the development and progression of T2DM and its complications [1, 9]. Insulin, secreted by *β* cells, plays crucial roles in many metabolic processes such as regulating the glucose uptake [1, 10]. Therefore, when insulin secretion is impaired, glucose uptake will also be affected. Persistent hyperglycemia will lead to the overproduction of reactive oxygen species, which can cause oxidative damage to deoxyribonucleic acid (DNA), proteins, and lipids [1, 10, 11]. Because the mitochondria are in proximity to the sources of reactive oxygen species, oxidative stress often leads to mitochondrial damage, resulting in mitochondrial dysfunction. An increasing number of studies have shown that in obese and T2DM patients, reactive oxygen species can aggravate the insulin resistance status and interfere with the insulin signaling pathway through impairing the mitochondrial ability to oxidize fat [12, 13]. Meanwhile, the abnormality in mitochondrial functions secondary to oxidative stress is one of the mechanisms leading to T2DM and T2DM-related complications [11].

Coenzyme Q10 is a lipid-soluble nutrient widely present in living cells. Coenzyme Q10, as an effective antioxidant, can scavenge free radicals and protect cells from oxidation. Recent studies have found that T2DM patients have significantly lower levels of coenzyme Q10 than healthy people [1, 14–16], which indicates that coenzyme Q10 deficiency may reduce the organism's ability to counter hyperglycemia-induced oxidative stress in T2DM [17, 18]. This suggests that coenzyme Q10 plays an important role in the pathogenesis of T2DM [19]. Therefore, exogenous coenzyme Q10 supplements may improve the oxidative stress-induced abnormalities in mitochondrial functions, thereby bettering glycemic control in patients with T2DM [20].

Therefore, the purpose of this paper is to review the available randomized controlled trials (RCTs) to evaluate the effectiveness of coenzyme Q10 for T2DM. Although systematic reviews and meta-analyses of the effects of coenzyme Q10 on the metabolic profile of diabetes mellitus [21] and diabetes-related biomarkers [22] have been performed, the analysis in 2015 only showed that coenzyme Q10 may reduce triglyceride levels, while the analysis in 2016 only included RCTs before 2014. Over time, more RCTs about coenzyme Q10 were published between 2014 and 2018. Therefore, the results of systematic reviews and meta-analyses need to be updated. This systematic review and meta-analysis is a registered review with protocol (CRD42018088474) in PROSPERO, which is aimed at evaluating the effects of coenzyme Q10 on T2DM.

## 2. Materials and Methods

### 2.1. Protocol

Study selection, assessment of eligibility criteria, data extraction, and statistical analyses were performed based on a predefined protocol registered on PROSPERO (CRD42018088474) (see Supplementary Materials available here).

### 2.2. Search Strategy and Study Selection

Records of coenzyme Q10 supplementation in T2DM were identified through a systematic literature search from the China National Knowledge Infrastructure (CNKI) Databases, Chinese Biomedical Database (CBM), Cochrane Library (until Issue 2, 2018), Web of Science, Embase, Wan Fang Database (Chinese Ministry of Science and Technology), PubMed, MEDLINE Complete, ClinicalTrials.gov, and Chinese Science and Technology Periodical Database (VIP), from their inception to February 2018. The search terms included Coenzyme Q10, CoQ 10, CoQ10, ubidecarenone, co-enzyme Q10, ubiquinone Q10, Bio-Quinone Q10, ubiquinone 50, ubisemiquinone radical, ubisemiquinone, ketosis-resistant diabetes mellitus, non-insulin-dependent diabetes mellitus, stable diabetes mellitus, and type 2 diabetes mellitus. For example, the search strategy for PubMed is presented in Table 1.

Studies meeting the inclusion criteria were included in this review (see Box 1).

### 2.3. Data Extraction

Three reviewers (Shi-ying Zhang, Kai-lin Yang, and Liu-ting Zeng) independently selected the studies and extracted the data from the studies. Disagreements were resolved by discussion of five reviewers (Shi-ying Zhang, Kai-lin Yang, Liu-ting Zeng, Xiao-he Wu, and Hui-yong Huang). We first reviewed the titles and abstracts of each of the studies and excluded the articles that do not meet the eligibility criteria. Then, we assessed the full texts of studies that meet the criteria. A customized form was used to record authors, year of publication, intervention, control group, outcomes, AEs, and duration.

If there is missing information in the article, reviewers would attempt to contact the authors to obtain the data or impute the missing data according to the Cochrane Handbook 5.1.0 [23]. If *P* < 0.05, the missing standard deviations would be imputed by a *P* value; if *P* > 0.05 or *P* = NS, it would be imputed by using the average of candidate standard deviations [23].

### 2.4. Study Quality Assessment

We assessed the risk of bias by using the risk of a bias assessment tool based on the Cochrane Handbook [24]. The criteria consist of 7 items: random sequence generation, allocation concealment, blinding of participants and personnel, blinding of outcome assessment, incomplete outcome data, selective outcome reporting, and other sources of bias. The study quality assessment was performed by three reviewers (Shi-ying Zhang, Kai-lin Yang, and Liu-ting Zeng) independently. Disagreements were resolved by consensus among all five reviewers (Shi-ying Zhang, Kai-lin Yang, Liu-ting Zeng, Xiao-he Wu, and Hui-yong Huang).

### 2.5. Statistical Analysis

The data were analyzed by RevMan 5.3 software. The dichotomous variable measure was summarized by risk ratio (RR) with a 95% confidence interval (CI). The continuous outcomes underwent meta-analysis using mean differences (MD) and 95% CI. Heterogeneity among studies was assessed using Cochrane's *Q* and *I* ^2^ statistic [25]. The fixed effect model would be used when *P* > 0.1 and *I* ^2^ < 50%. We would explore the reasons for heterogeneity, perform the subgroup analysis, or use the random effects model when *P* < 0.1 and *I* ^2^ > 50%.

## 3. Results

### 3.1. Results of the Search

**Figure 1:**
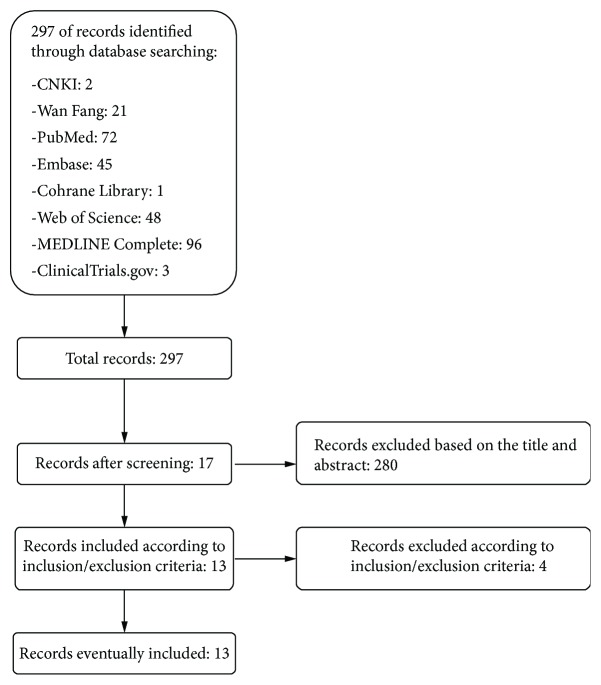
Flow diagram of searching and article selection.

Two hundred ninety-seven articles were found in the initial search; two hundred eighty of them were excluded based on the title and abstract and seventeen of them were retrieved for more detailed evaluation. Finally, thirteen studies were included in this systematic review and meta-analysis, and four were excluded (Figure 1).

### 3.2. Description of Included Trials and Risk of Bias in Included Studies

Thirteen RCTs with 765 participants met the inclusion criteria. All of them were parallel-group RCTs. In Playford et al.'s [26] and Chew et al.'s research [27], there were two trial groups and two control groups. According to the Cochrane Handbook 5.1.0, we split the shared trial and control groups into two groups with a smaller sample size [23] and include the four reasonably independent comparisons [Playford 2002a (coenzyme Q10 vs. placebo), Playford 2002b (coenzyme Q10 vs. Fenofibrate), Playford 2002c (coenzyme Q10 + fenofibrate vs. placebo), and Playford 2002d (coenzyme Q10 + fenofibrate vs. fenofibrate) and Chew 2008a (coenzyme Q10 vs. placebo), Chew 2008b (coenzyme Q10 vs. fenofibrate), Chew 2008c (coenzyme Q10 + fenofibrate vs. placebo), and Chew 2008d (coenzyme Q10 + fenofibrate vs. fenofibrate)]. Study characteristics are presented in Table 2.

**Figure 2:**
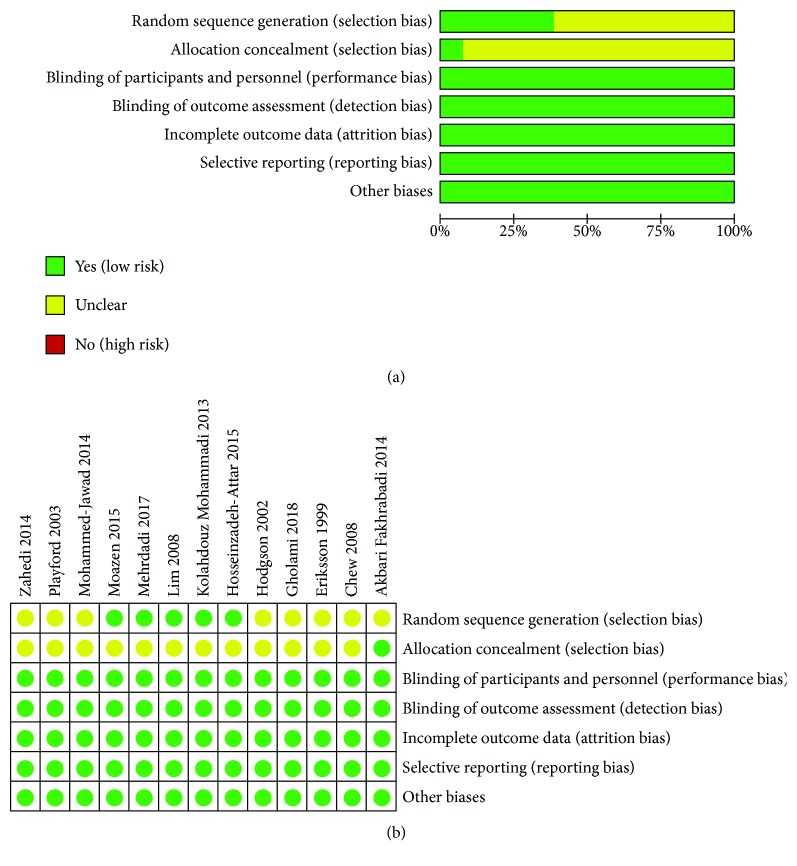
(a, b) The risk of bias.

With regard to the selection bias, eight RCTs [19, 26–32] did not describe the randomization procedures, and twelve RCTs [19, 26–28, 30–37] did not describe an acceptable method of allocation concealment; thus, we thought that their risk of bias is unclear. One RCT [33] used block randomization, three RCTs [34, 36, 37] utilized permuted random block allocation, and one RCT used a website [35] to conduct randomization; we therefore rated them as having a low risk of bias. One RCT [29] describes an acceptable method of allocation concealment, and it was rated as having a low risk of bias. As for the performance bias and detection bias, two trials were unclear [32, 33], but they used objective measures (e.g., HbA1c, fasting insulin, and fasting glucose) and the outcome is not likely to be influenced by the lack of blinding, while the remaining three studies used blinding; thus, we gave a low risk of bias for all. None of trials missed data and incompletely reported the outcomes; therefore, we gave a low risk of bias. Other sources of bias were at low risk in all of the included studies. A graphical summary of the risks of bias assessment is presented in Figure 2.

### 3.3. Primary Outcomes

#### 3.3.1. Glycosylated Hemoglobin

**Figure 3:**
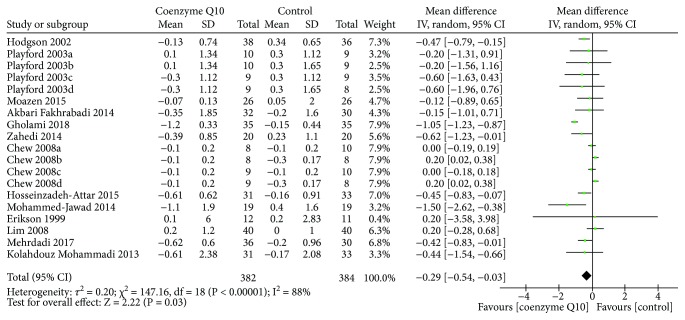
Glycosylated hemoglobin.

All of the RCTs reported the HbA1c at the end of treatment. Due to the high heterogeneity (*τ* ^2^ = 0.20, *I* ^2^ = 88%, *P* < 0.00001), the random effects model was used. As shown in Figure 3, coenzyme Q10 may decrease the HbA1c compared with the control group (WMD −0.29; 95% CI −0.54, −0.03; *P* = 0.03).

#### 3.3.2. Fasting Blood Glucose

**Figure 4:**
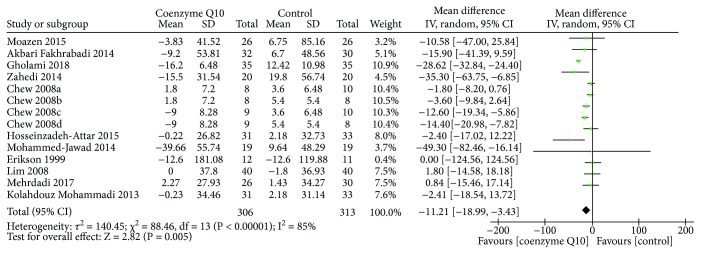
Fasting blood glucose.

Ten RCTs [19, 27, 29–37] reported the fasting blood glucose. The random effects model was utilized because of the high heterogeneity (*τ* ^2^ = 140.45, *I* ^2^ = 85%, *P* < 0.00001). As shown in Figure 4, coenzyme Q10 may decrease the fasting blood glucose compared with the control group (WMD −11.21; 95% CI −18.99, −3.43; *P* = 0.005).

#### 3.3.3. Fasting Insulin

**Figure 5:**
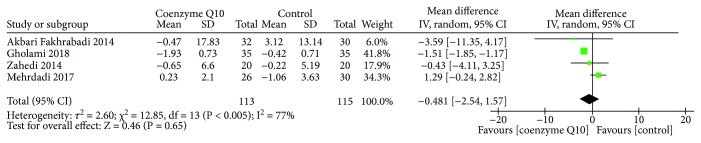
Fasting insulin.

Four RCTs [29–31, 36] reported the fasting insulin. The random effects model was used due to the high heterogeneity (*τ* ^2^ = 2.60, *I* ^2^ = 77%, *P* = 0.005). The result showed that there is no statistically significant difference between the coenzyme Q10 group and control group in adjusting insulin (WMD −0.48; 95% CI −2.54, 1.57; *P* = 0.65) (Figure 5).

### 3.4. Secondary Outcomes

#### 3.4.1. Homeostasis Model Assessment of Insulin Resistance

**Figure 6:**
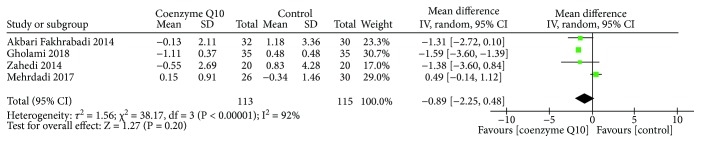
Homeostasis model assessment of insulin resistance.

Four RCTs [29–31, 36] reported HOMA-IR. Due to the high heterogeneity (*τ* ^2^ = 1.56, *I* ^2^ = 92%, *P* < 0.00001), the random effects model was utilized. The results showed that in improving the HOMA-IR, the difference between the coenzyme Q10 group and the control group was not statistically significant (MD −0.89; 95% CI −2.25, 0.48; *P* = 0.20) (Figure 6).

#### 3.4.2. Blood Lipids

**Figure 7:**
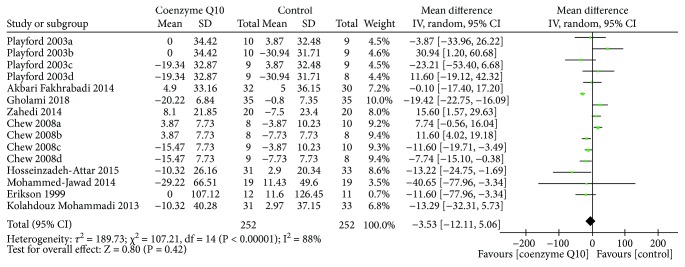
Total cholesterol.

Nine RCTs [19, 26, 27, 29–32, 34, 37] reported TC. The random effects model was utilized because of the high heterogeneity (*τ* ^2^ = 189.73, *I* ^2^ = 87%, *P* < 0.00001). In this index, there is also not strong evidence that confirms which one is better because there was no statistical difference (WMD −3.53; 95% CI −12.11, 5.08; *P* = 0.42) (Figure 7).

**Figure 8:**
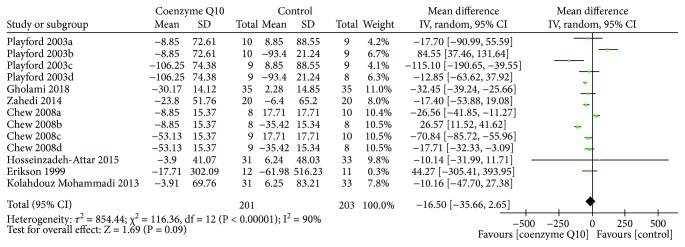
Triglyceride.

Seven RCTs [19, 26, 27, 30, 31, 34, 37] reported TG. The random effects model was utilized due to the high heterogeneity (*τ* ^2^ = 854.44, *I* ^2^ = 90%, *P* < 0.00001). In this index, there is also not strong evidence that confirms which one is better (WMD −16.50; 95% CI −35.66, 2.65; *P* = 0.09) (Figure 8).

**Figure 9:**
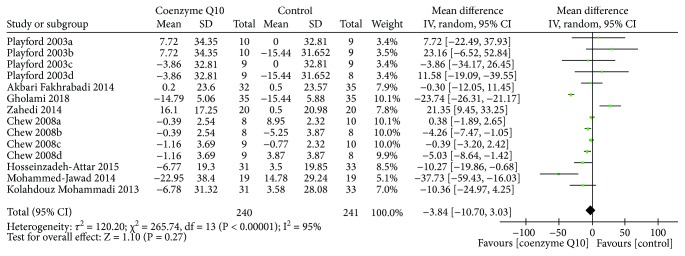
LDL-C.

Eight RCTs [26, 27, 29–32, 34, 37] reported LDL-C. Due to the high heterogeneity (*τ* ^2^ = 120.20, *I* ^2^ = 95%, *P* < 0.00001), the random effects model was used. The results showed that in decreasing the LDL-C, the difference between the coenzyme Q10 group and the control group was not statistically significant (WMD −3.84; 95% CI −10.70, 3.03; *P* = 0.27) (Figure 9).

**Figure 10:**
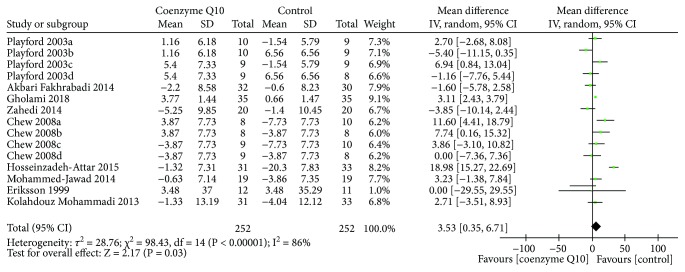
HDL-C.

Nine RCTs [19, 26, 27, 29–32, 34, 37] reported HDL-C. The random effects model was used due to the high heterogeneity (*τ* ^2^ = 28.76, *I* ^2^ = 86%, *P* < 0.00001). As shown in Figure 10, coenzyme Q10 may increase HDL-C levels compared to the control group (WMD 3.53; 95% CI 0.35, 6.71; *P* = 0.03).

#### 3.4.3. Adiponectin

**Figure 11:**
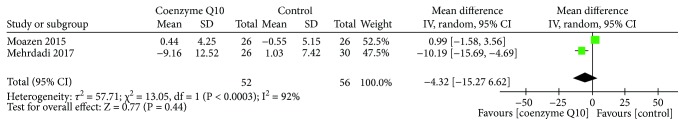
Adiponectin.

Two RCTs [33, 36] reported adiponectin. Due to the high heterogeneity (*τ* ^2^ = 57.71, *I* ^2^ = 92%, *P* = 0.0003), we used the random effects model. The results showed that there was no statistically significant difference between the coenzyme Q10 group and the control group in improving the adiponectin (WMD −4.32; 95% CI −15.27, 6.62; *P* = 0.44) (Figure 11).

### 3.5. Adverse Events

None of RCTs reported AEs.

## 4. Discussions

This study is a systematic review and meta-analysis that regards the effects of coenzyme Q10 on T2DM patients. It synthesizes the results from 13 RCTs (including the newest RCTs [29–34, 36] after 2014) involving 765 participants to draw an overall conclusion. Although significant differences between groups were found for some outcomes, the available evidence shows that coenzyme Q10 may improve the glycemic control (decreasing the HbA1c and fasting blood glucose) and blood lipids (decreasing TG and increasing HDL-C), suggesting that coenzyme Q10 may assist glycemic control and protect the cardiovascular system. However, these findings should be interpreted with caution although it seems like that they are prospective. The unclear risk of bias for selection bias (random sequence generation and allocation concealment), the small number of patients, and the high heterogeneity limited the promotion of the results. In addition, the lack of statistical significance of fasting insulin, HOMA-IR, TC, LDL-C, and adiponectin does not equate to no medical significance. Instead, it may mean that coenzyme Q10 may be the safer or cheaper treatment options.

Coenzyme Q10 deficiency, particularly ubiquinol (the reduced form of coenzyme Q10) deficiency, is often observed among patients with T2DM. The ubiquinol/ubiquinone ratio is often utilized as an indicator to react to the body's oxidative stress [38]. Decreased ubiquinol levels are often accompanied by increased ubiquinone levels, suggesting that there is an ineffective conversion between ubiquinone and ubiquinol. It also indicates that the body's ability to scavenge free radicals is reduced. Meanwhile, the impaired conversion of ubiquinone to ubiquinol is often found in many diseases [39]. Román-Pintos et al. [15] found that coenzyme Q10 levels in T2DM patients were significantly lower than those in healthy people, while their MDA levels were significantly higher than those in healthy one. Furthermore, the ubiquinone/ubiquinol ratio in T2DM patients was continuously higher than that in normal heathy people throughout the day after breakfast, which indicates that postprandial hyperglycemia is associated with increased oxidative stress [16]. Meanwhile, exogenous coenzyme Q10 supplementation can increase over 31% of the activity of succinate dehydrogenase in patients with T2DM [15]. The mitochondrial succinate dehydrogenase is a flavoprotein in the mitochondrial inner membrane which can donate electrons to coenzyme Q10. Since the Krebs cycle relies on succinate dehydrogenase and NADH dehydrogenase, it can be inferred that appropriate coenzyme Q10 level is beneficial for this cycle [40]. Therefore, based on the evidence above, the abnormalities in mitochondrial functions secondary to oxidative stress in T2DM patients may be potentially relieved by restoring the coenzyme Q10 level through exogenous coenzyme Q10 supplementation, ultimately improving the glycemic control.

None of RCTs reported information about AEs, which means that there is currently a lack of reports on AEs. However, this does not mean that the intervention of coenzyme Q10 is safe [41]. Thus, although, based on current evidences, we consider that coenzyme Q10 is a relatively safe treatment, we cannot assure it. Future clinical trials are required to report AEs with more explanations [42].

Compared with previous reviews [21, 22], the strengths of this systematic review and meta-analysis are that it is strictly conducted in accordance with the protocol registered on PROSPERO and it assesses more outcomes. It also included seven new RCTs [29–34, 37] after 2014. Of course, this systematic review and meta-analysis also has limitations, including but not limited to the number of patients participated which is only 765 and the heterogeneity of some outcomes. Such heterogeneity confounds the interpretation of statistical findings. The heterogeneity may come from the potential discrepancies in the pharmacological effects of various coenzyme Q10 preparations which may result from different standardizations of the coenzyme Q10 manufacturing process, dosage, duration of treatment, units of laboratory tests, and races of the selected patients or other places. Therefore, the random effects model was adopted, although it cannot completely eliminate heterogeneity. Meanwhile, the study duration is generally short-to-medium term (mostly 12 weeks), and the long-term efficacy of coenzyme Q10 is temporarily uncertain. Furthermore, in the included studies, the dose of CoQ10 in the experimental group was not necessarily the same, which could lead to differences in the effect of lowering blood glucose; the difference in dosage makes it difficult to determine the minimum effective dose of coenzyme Q10. Finally, due to the fact that none of the trials reported AEs, the safety of coenzyme Q10 should be interpreted with caution. In the future, more similar high-quality randomized controlled trials are needed to amend the results of this systematic review and meta-analysis.

## 5. Conclusion

Coenzyme Q10 may assist glycemic control, decrease TG, and improve HDL-C in patients with T2DM. However, the limitations in RCTs, including small sample sizes and short duration, make the result be interpreted cautiously. The benefits from long-term treatment of coenzyme Q10 beyond 6 months remain to be defined by future studies. Meanwhile, more randomized, double-blind, large-sample-size trials of coenzyme Q10 for T2DM are needed in the future to validate or revise the result of this work.

## Figures and Tables

**Box 1:**
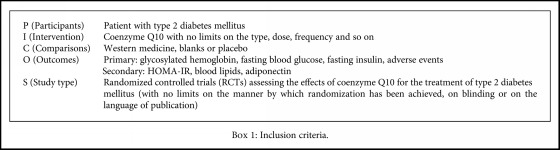
Inclusion criteria.

**Table 1:** Search strategy for PubMed.

Database	Search strategy
PubMed	(Coenzyme Q10 OR CoQ 10 OR CoQ10 OR ubidecarenone OR co-enzyme Q10 OR ubiquinone Q10 OR Bio-Quinone Q10 OR 2,3-dimethoxy-5-methyl-6-decaprenylbenzoquinone OR ubiquinone 50 OR ubisemiquinone radical OR Q-ter OR ubisemiquinone OR coenzyme Q10, (Z,Z,Z,Z,Z,Z,E,E,E)-isomer OR coenzyme Q10, ion (1-), (all-E)-isomer) AND (Type 2 diabetes mellitus OR Diabetes Mellitus, Noninsulin-Dependent OR Diabetes Mellitus, Ketosis-Resistant OR Diabetes Mellitus, Ketosis Resistant OR Ketosis-Resistant Diabetes Mellitus OR Diabetes Mellitus, Non Insulin Dependent OR Diabetes Mellitus, Non-Insulin-Dependent OR Non-Insulin-Dependent Diabetes Mellitus OR Diabetes Mellitus, Stable OR Stable Diabetes Mellitus OR Diabetes Mellitus, Type II OR NIDDM OR Diabetes Mellitus, Noninsulin Dependent OR Diabetes Mellitus, Maturity-Onset OR Diabetes Mellitus, Maturity Onset OR Maturity-Onset Diabetes Mellitus OR Maturity Onset Diabetes Mellitus OR MODY OR Diabetes Mellitus, Slow-Onset OR Diabetes Mellitus, Slow Onset OR Slow-Onset Diabetes Mellitus OR Type 2 Diabetes Mellitus OR Noninsulin-Dependent Diabetes Mellitus OR Noninsulin Dependent Diabetes Mellitus OR Maturity-Onset Diabetes OR Diabetes, Maturity-Onset OR Maturity Onset Diabetes OR Type 2 Diabetes OR Diabetes, Type 2 OR Diabetes Mellitus, Adult-Onset OR Adult-Onset Diabetes Mellitus OR Diabetes Mellitus, Adult Onset) AND (randomized controlled trial [pt] OR controlled clinical trial [pt] OR placebo [tiab] OR drug therapy [sh] OR trial [tiab] OR groups [tiab] OR clinical trials as topic [mesh: noexp] OR Clinical Trial OR random^∗^ [tiab] OR random allocation [mh] OR single-blind method [mh] OR double-blind method [mh] OR cross-over studies) NOT (animals [mh] NOT humans [mh])

**Table 2:** The characteristics of the included studies.

Study	Sample size		Intervention		Relevant outcomes	Mean age (years)		Duration
	Trial group	Control group	Trial group	Control group		Trial group	Control group	
Hodgson et al. [28]	38	36	Coenzyme Q10 200 mg or coenzyme Q10 200 mg + fenofibrate 200 mg	Placebo or fenofibrate 200 mg	HbA1c	52 ± 6.41	54.4 ± 9.88	12 weeks
Playford et al. [26]	38	35	Coenzyme Q10 200 mg or coenzyme Q10 200 mg + fenofibrate 200 mg	Placebo or fenofibrate 200 mg	HbA1c, TG, TC, HDL-C, LDL-C	52.7 ± 9.80	54.1 ± 13.7	12 weeks
Moazen et al. [33]	26	26	Coenzyme Q10 200 mg	Microcrystalline cellulose (placebo)	Fasting blood glucose, HbA1c, adiponectin	50.67 ± 7.01	52.79 ± 7.66	8 weeks
Akbari Fakhrabadi et al. [29]	32	30	Coenzyme Q10 200 mg	Microcrystalline cellulose (placebo)	Fasting blood glucose, fasting insulin, HbA1c, HOMA-IR, TC, HDL-C, LDL-C	56.7 ± 6.4	54.8 ± 6.7	12 weeks
Gholami et al. [30]	35	35	Coenzyme Q10 100 mg	Cellulose acetate 100 mg	Fasting blood glucose, fasting insulin, HbA1c, HOMA-IR, TC, TG, HDL-C, LDL-C	52.97 ± 1.04	53.68 ± 1.14	12 weeks
Zahedi et al. [31]	20	20	Coenzyme Q10 150 mg	Maize starch 150 mg	Fasting blood glucose, fasting insulin, HbA1c, HOMA-IR, TC, TG, HDL-C, LDL-C	53.5 ± 9.7	58.8 ± 9.6	12 weeks
Chew et al. [27]	36	33	Coenzyme Q10 200 mg or coenzyme Q10 200 mg + fenofibrate 160 mg	Placebo or fenofibrate 160 mg	Fasting blood glucose, HbA1c, TC, TG, HDL-C, LDL-C	62.22 ± 7.41	63.57 ± 8.09	24 weeks
Hosseinzadeh-Attar et al. [34]	31	33	Coenzyme Q10 200 mg	Maize starch 200 mg	Fasting blood glucose, HbA1c, TC, TG, HDL-C, LDL-C	45.2 ± 7.6	47.1 ± 8.3	12 weeks
Mohammed-Jawad et al. [32]	19	19	Coenzyme Q10 200 mg + antidiabetic drugs	Antidiabetic drugs	Fasting blood glucose, HbA1c, TC, HDL-C, LDL-C	49.37 ± 6.65	51.63 ± 8.13	8 weeks
Eriksson et al. [19]	12	11	Coenzyme Q10 200 mg	Placebo 200 mg	Fasting blood glucose, HbA1c, TC, TG, HDL-C	65 ± 17.32	64 ± 23.22	24 weeks
Lim et al. [35]	40	40	Coenzyme Q10 200 mg	Placebo 200 mg	Fasting blood glucose, HbA1c	54 ± 9	53 ± 9	12 weeks
Mehrdadi et al. [36]	26	30	Coenzyme Q10 200 mg	Placebo 200 mg	Fasting blood glucose, fasting insulin, HbA1c, adipolin, HOMA-IR	46 ± 7	48 ± 8	12 weeks
Kolahdouz Mohammadi et al. [37]	31	33	Coenzyme Q10 200 mg	Placebo 200 mg	Fasting blood glucose, HbA1c, TG, TC, HDL-C, LDL-C	45.23 ± 7.64	47.18 ± 8.31	12 weeks

## Supplementary Materials

Supplementary Materials CRD42018088474.pdf is the protocol of this systematic review and meta-analysis; PRISMA 2009 checklist.doc is the PRISMA checklist.
